# The effect of optical substrates on micro-FTIR analysis of single mammalian cells

**DOI:** 10.1007/s00216-012-6521-6

**Published:** 2012-11-14

**Authors:** Katia Wehbe, Jacob Filik, Mark D. Frogley, Gianfelice Cinque

**Affiliations:** Diamond Light Source, Harwell Science and Innovation Campus, Didcot, Oxfordshire OX11 0DE UK

**Keywords:** Single-cell analysis, Synchrotron radiation IR microspectroscopy, IR optical substrates, Transmission, Reflection, PCA

## Abstract

The study of individual cells with infrared (IR) microspectroscopy often requires living cells to be cultured directly onto a suitable substrate. The surface effect of the specific substrates on the cell growth—viability and associated biochemistry—as well as on the IR analysis—spectral interference and optical artifacts—is all too often ignored. Using the IR beamline, MIRIAM (Diamond Light Source, UK), we show the importance of the substrate used for IR absorption spectroscopy by analyzing two different cell lines cultured on a range of seven optical substrates in both transmission and reflection modes. First, cell viability measurements are made to determine the preferable substrates for normal cell growth. Successively, synchrotron radiation IR microspectroscopy is performed on the two cell lines to determine any genuine biochemically induced changes or optical effect in the spectra due to the different substrates. Multivariate analysis of spectral data is applied on each cell line to visualize the spectral changes. The results confirm the advantage of transmission measurements over reflection due to the absence of a strong optical standing wave artifact which amplifies the absorbance spectrum in the high wavenumber regions with respect to low wavenumbers in the mid-IR range. The transmission spectra reveal interference from a more subtle but significant optical artifact related to the reflection losses of the different substrate materials. This means that, for comparative studies of cell biochemistry by IR microspectroscopy, it is crucial that all samples are measured on the same substrate type.

**Figure:**
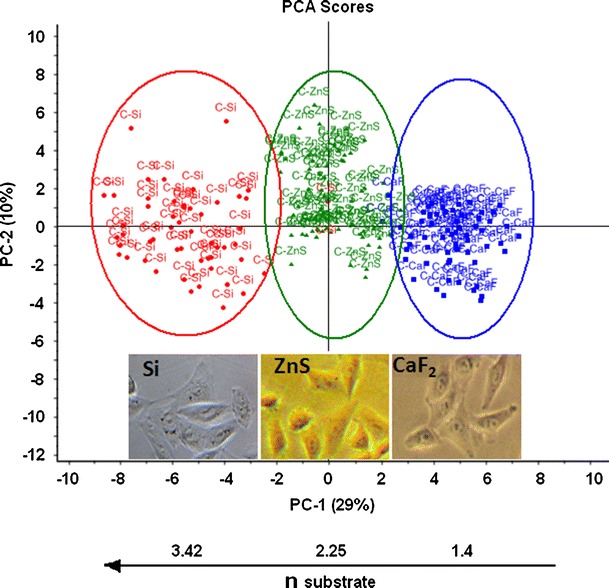
Cell separation by PCA due to the refractive index of the substrate used, revealing transmission artifact.

Cell separation by PCA due to the refractive index of the substrate used, revealing transmission artifact.

## Introduction

The use of infrared (IR) spectroscopy for studying biological cells is nowadays a wide and active area of research. Specifically, synchrotron radiation (SR) IR microspectroscopy, giving the high spatial resolution and signal-to- noise necessary for single-cell analysis, has proved to be an ideal tool for investigating the biochemical composition of biological samples at the microscopic and molecular scale [1–3]. It has been shown that synchrotron-based Fourier-transform infrared (FTIR) microspectroscopy has no cytotoxic effects on examined cells as no detectable biochemical changes between control and exposed cells have been found despite the increased power density of the SR light [4]. IR spectral differences have been reported between cancerous and normal cells [5, 6], between cells in different growth stages [7–9], or as an effect of drugs on cells [10–13]. There is no unique substrate used in all these studies, and no comparison has been reported in literature of which one is more suitable for cell growth and IR analysis. If the substrate has any influence on the measurements, either through biochemical or morphological changes in the cells, or through systematic variation in the spectral data via optical artifacts, it is clear that these effects would need to be identified and that standardization would be required to allow direct comparison between different works.

Cell adhesion on the substrate is a key aspect for cellular morphology, proliferation, and function. Poly-l-lysine, laminin, fibronectin, collagen, and other components are used as substrate coatings to enhance cell adhesion, but they may interfere with the cell spectra. A study on cancerous cells done by Draux et al. [14] compared three substrates used for Raman microspectroscopy (Quartz, ZnSe, and CaF_2_). This study revealed that quartz and CaF_2_ were much better for cell growth than ZnSe, which showed a very weak cell adherence due to its toxicity. Another study by Meade et al. [15] on keratinocytes compared MirrIR and quartz substrates by IR and Raman spectroscopy but using three different coatings (laminin, fibronectin, and gelatin) for cell adhesion. This study showed that functional changes regarding proliferation and viability as well as spectral changes were induced and could influence the spectroscopic measurement. In a previous study by Carter et al. [16], Si_3_N_4_ was shown to be suitable for cell growth for FTIR and XRF analysis. There are no reports comparing a wide range of IR optical substrates and studying their direct effect on cell growth.

In general, metal substrates are used for reflection measurements and inorganic crystals for transmission. These materials all have different surface chemistries, some being highly biocompatible and others, potentially toxic. This raises a simple question: Do the different substrates interfere chemically with the cell growth? The effect from these materials with biological samples is likely to be minimal when tissue sections or cells are deposited on the surface but may be crucial when cells are grown for several hours or days and then fixed before IR analysis. There may be an interaction of the substrate material with the culture medium or even the fixatives. Thus, the underlying chemical interface layer could play an important role for viability and morphology of cells growing on these substrates.

The reflection measurement geometry has the advantages of a stronger absorption (due to the doubling of the path length) and cost-effective substrates. MirrIR- and aluminum-coated glass slides are typically used for IR reflection (also known as transflectance) measurements when studying cells. MirrIR slides, glass slides with reflective multilayer coating (Ag/SnO_2_), are especially popular because samples can be examined by conventional light microscopy in transmission and then scanned by IR in reflection mode.

In the transmission geometry, a wide variety of substrates can be used. These optical materials have different spectral ranges, e.g., CaF_2_ (0.35 to 10 μm wavelength) versus Si (1.2 to 15 μm wavelength) and also different refractive indices with associated reflection losses at the substrate interfaces.

The use of these two IR geometries, each with different substrates with different chemical and optical properties, raises further questions such as: Is there any difference of IR spectra between transmission and reflection measurements for the same type of cells, and do the cells grow well and in the same way on all of these substrates? To answer these questions, in this study, a wide range of IR optical substrates, CaF_2_, Si, ZnSe, BaF_2_, and ZnS for transmission and MirrIR and Al slides for reflection were compared using two cell lines. No additional coatings were applied to the substrates; the cells were grown directly on the surface to study the immediate effect of each IR optical substrate on the cell growth and biochemistry. The two cell lines selected are both adherent cell lines but from different origins. Chinese hamster ovary cells (CHO-K1) are epithelial-like and one of the most used mammalian cell lines in biological and medical research. Colorectal adenocarcinoma cell line (DLD1) is a human colon cancer cell line used as an example of cancerous cells.

## Materials and methods

### Cell preparation

Cells were cultured in plastic culture flasks (polystyrene) using Hamm’s F12 medium (Sigma-Aldrich) for CHO-K1 and RPMI 1640 medium (Gibco) for DLD1. Both media were supplemented with 10 % FBS, 1 % l-glutamine, and 1 % penicillin/ streptomycin (all from Gibco, Invitrogen). Cells were maintained in a humidified atmosphere in a 37 °C incubator supplied with 5 % CO_2_. Before reaching confluence, cells were detached using trypsin–EDTA 0.25 % (Gibco) and then centrifuged. The pellet was collected, resuspended in culture media, and then seeded on different IR optical substrates for transmission, i.e., CaF_2_, Si, ZnSe, BaF_2_, and ZnS (Crystran, UK) and reflection measurements, i.e., MirrIR slides (Kevley Technologies, OH, USA) and Al slides (Thermofisher, UK). All substrates were cleaned with 70 % ethanol before being used for cell culture. Cells were seeded at a concentration of 5 × 10^4^ cells/ml of medium. After 48 h incubation, cells were washed with NaCl 0.9 % and fixed with 4 % formalin (Sigma-Aldrich) for 30 min, washed with distilled water, and then dried before analysis under the IR microscope. For further viability comparison on different substrates, other sets of cells were fixed with ice-cold acetone before staining for epifluorescence observation.

### Epifluorescence with DAPI and PI staining

For morphological and viability observation, parallel series of cells were stained with propidium iodide (PI) and 4′,6-diamidino-2-phenylindole (DAPI). After 48 h culture on different substrates, cells were rinsed with phosphate-buffered saline (PBS) 1×, fixed with ice-cold acetone at −20 °C for 10 min, and then washed with PBS. Cells were then equilibrated with 2× SSC (0.3 M NaCl, 0.03 M sodium citrate, pH 7.0; Gibco, Invitrogen). Cells were incubated with the dilute PI stain (Molecular probes, Invitrogen) for 1–5 min (500 nM solution of PI by diluting the 1 mg/ml corresponding to 1.5 mM stock solution 1:3,000 in 2× SSC). Cells were then rinsed three times in 2× SSC and mounted with the Prolong Gold antifade with DAPI reagent (Molecular probes, Invitrogen) and coverslipped. Samples were viewed using the fluorescence microscope (Zeiss Axio-imager M1) with the appropriate excitation/detection filters.

### FTIR data acquisition and analysis

Single CHO-K1 and DLD1 cells grown on IR optical substrates were analyzed in the mid-IR range (4,000–600 cm^−1^) on the (Bruker) Vertex 80 V FTIR spectrometer available at the IR Beamline B22 (MIRIAM) in Diamond Light Source, UK [17]. The spectra were measured using the LN_2_ cooled MCT broadband (>500 cm^−1^) detector (100 × 100 μm^2^ area), coupled to the Hyperion 3000 microscope and the SRIR source. The aperture size at the sample of 15 × 15 μm^2^ was used to collect spectra from single isolated cells at 4 cm^−1^ spectral resolution and 256 scans using the ×36 (0.5 NA) objective (matched with a ×36 condenser for transmission measurement).

All data acquisition was performed using OPUS 6.5 software (Bruker). Selection of spectra for data treatment was based on eliminating those with very weak absorbance (poor S/N ratio). Between 60 and 70 spectra were analyzed on each substrate for each cell line. Data analysis was performed in the Unscrambler X 10.1 software, taking second derivative spectra (Savitzky-Golay second order) to remove slowly varying baseline effects and then normalized using the standard normal variate (SNV). SNV is an analytical transformation applied to spectra to remove multiplicative interferences of scatter effects by centering and scaling each individual spectrum using only the data from that spectrum and not the mean spectrum of the set. Principal component analysis (PCA) was performed for each cell line population and then for both cell lines grouped together, using the nonlinear iterative partial least squares algorithm and leverage correction validation method.

Although the nucleic acids region (1,150–1,010 cm^−1^) could be identified in spectra from most of the substrates, the mid-IR spectral region (3,800–1,100 cm^−1^) excluding the CO_2_ region (2,400–2,100 cm^−1^) was used for PCA, since it includes most of the normal vibration modes of the common biological molecules (proteins, lipids, etc.). This choice allowed making a fair comparison between the substrates because the nucleic acid region can be strongly affected by the different spectral IR bandwidth of the materials (CaF_2_ cuts off at 1,000 cm^−1^, transmission range 0.35–10 μm wavelength).

## Results and discussion

### Cell growth and morphology observation

**Fig. 1:**
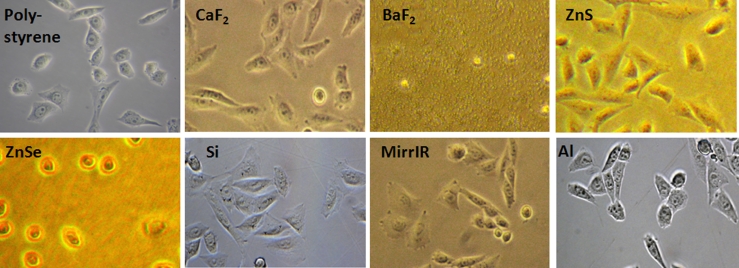
Visible light live cell images for CHO-K1 cells on polystyrene and IR optical substrates. Magnification ×20. Photos taken on Zeiss inverted microscope Axiovert in phase contrast mode for transparent substrates to visible light (polystyrene, CaF_2_, BaF_2_, ZnS, ZnSe, and MirrIR) and on Zeiss upright optical microscope Axioimager in reflection for opaque substrate (Si and Al). Note that cells did not grow on BaF_2_ surface while, on ZnSe, they are round-shaped and not fully developed

For cell growth comparison on different substrates, live cells were observed on an inverted microscope in phase contrast for the transparent substrates for visible light (CaF_2_, ZnSe, BaF_2_, ZnS, and MirrIR slides) and on an upright optical microscope for the reflective substrates (Si and Al slides) (Fig. 1). The images of cells grown on Si and Al were taken in reflection (not phase contrast), thus the images display a contour around the cells due to interference fringes. In order to assess cell viability on each substrate, the trypan blue exclusion method was used (trypan blue solution 0.4 %, Sigma-Aldrich). Viability test revealed that all substrates apart from BaF_2_ and ZnSe were suitable for cell growth (94 % of viable cells on CaF_2_, ZnS, Si, and Al slides, 94 % on the polystyrene culture flasks used as control, and 87 % on MirrIR slides, over 100 of cells were counted for each substrate to assess the viability). The reduced viability on MirrIR slides could be due to a partially toxic effect of the silver coating. Meade’s study [15] on keratinocytes grown on MirrIR with different types of added coating showed that the Ag/SnO_2_ coating on the reflective surface of the MirrIR plays a role in the cellular attachment, and this could be a result of surface roughness on the nanometer scale. In another study done by Mrkisich et al. on mammalian cell attachment on transparent films of gold and silver, it has been reported that inorganic silver salts may be released from the substrate and are toxic to cells [18].

**Fig. 2:**
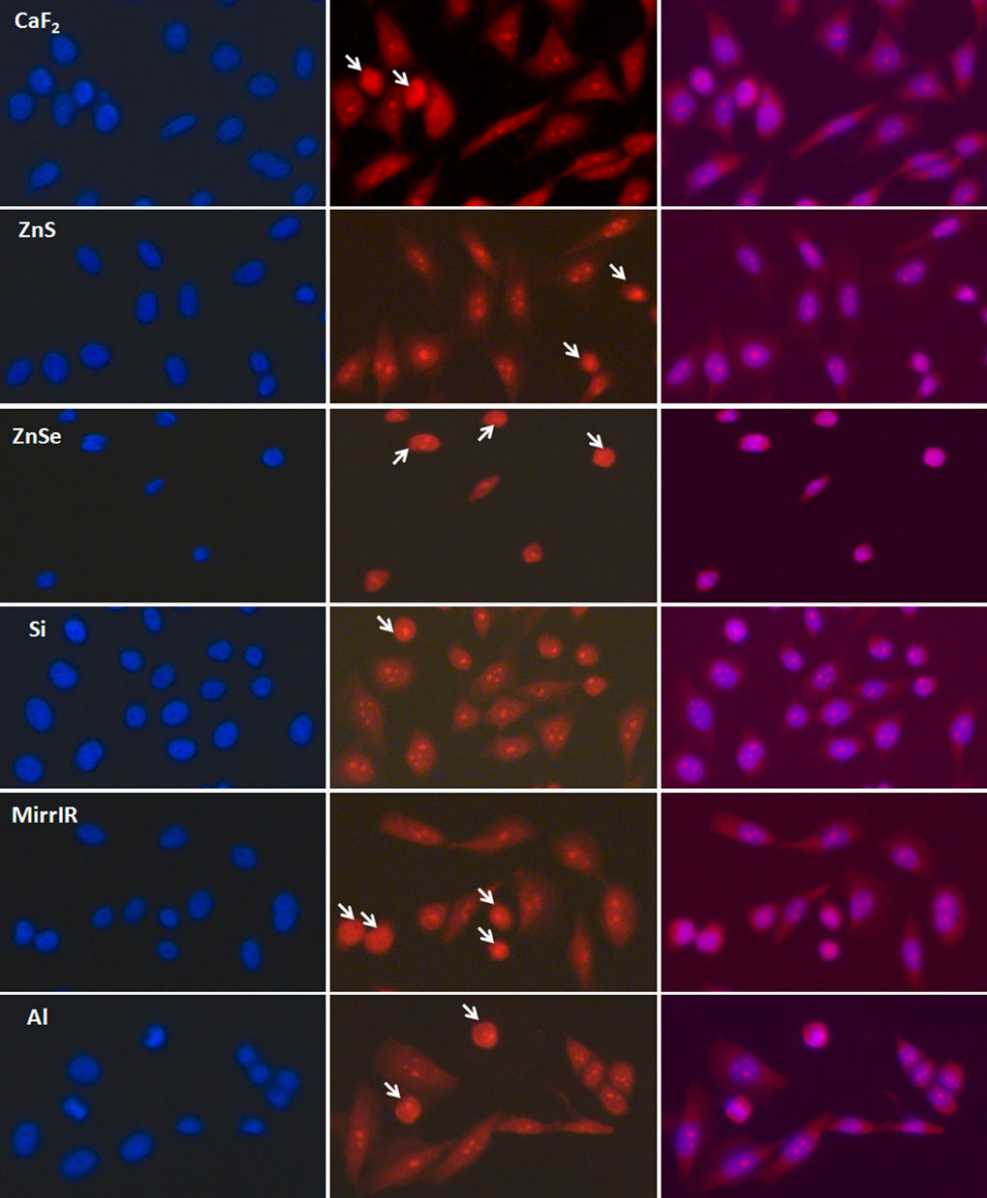
Epifluorescence observation for fixed CHO-K1 cells stained with PI and DAPI. Magnification ×20. Images of cells, taken on Zeiss Axioimager microscope, stained with DAPI (*first column*), PI (*second column*) and the combined image for the two dyes (*last column*). *Arrows* show the dead cells with condensed nucleus with the PI staining

BaF_2_ did not prove to be a good substrate to grow cells on, most likely due to its chemical toxicity in combination with its partial water solubility. This last effect caused the low optical image quality in Fig. 1. Cells grown on ZnSe substrate had a problem attaching to its surface (viability less than 30 %) and did not spread well. This phenomenon was also noticed in the Draux study [14] as, according to their results, ZnSe is toxic to cells. Cell morphology and viability were further assessed by epifluorescence observation after acetone fixation and PI and DAPI staining. PI (excitation, 535 nm; emission, 617 nm) is commonly used for identifying dead cells in a population; it binds to nucleic acids. DAPI (excitation, 358 nm; emission, 461 nm) is also a popular nuclear counterstain, and it stains specifically the nuclei with no cytoplasmic labeling. Cells observed in epifluorescence (Zeiss Axio-imager M1) with these two fluorescent dyes were also counted for viability. Dead cells showed a condensed nucleus with the PI staining (Fig. 2) compared with viable cells which have proper cytoplasm and nucleus stain distribution. PI dye is membrane-impermeant and generally excluded from viable cells, but it has been shown that, in fixed cells [19–21], it stains both cytoplasm and nucleus. However, PI is still able to differentiate between dead and live cells by showing condensed nucleus in dead cells if examined immediately after staining. The nuclei of dead cells show stronger red fluorescence due to higher PI absorbance. This could be also illustrated in the images with both dyes merged together where dead cells show nucleus in strong magenta color due to overlapping of DAPI and PI. Results from epifluorescence observation confirmed the same percentage of viability compared with the trypan blue exclusion method on each substrate. Moreover, results showed that the morphology was similar on all substrates except for the ZnSe where the cells showed a more rounded shape due to their non-attachment and development problems. However, it is noteworthy to say that, after repeating the experiment three times, cells grown on CaF_2_ and ZnS were the most similar in morphology to the cells grown in culture flasks.

### IR microspectroscopy of cells on different substrates

**Fig. 3:**
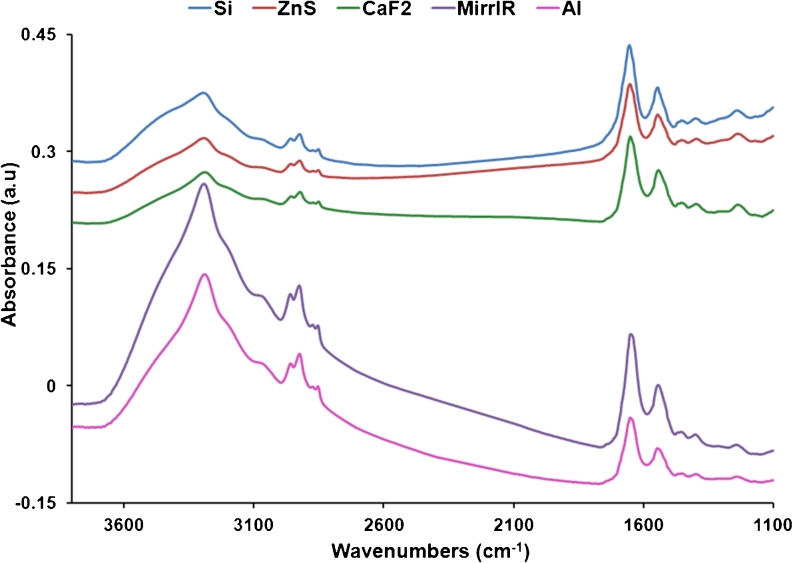
Total absorbance raw average spectra for CHO-K1 and DLD1 cells on each IR optical substrate. Spectra are offset for clarity. There is a clear difference in ratio of the high wavenumber region (3,600–2,800 cm^−1^) with respect to the low wavenumber region (1,700–1,100 cm^−1^) between T and R spectra

Using the IR microscope MCT detector, single-cell spectra were acquired using SRIR for both CHO-K1 and DLD1 cells on different IR optical substrates. Spectra of samples on ZnSe were not measured since cells did not proliferate on this substrate. In general, before any data treatment, the average spectra in transmission (CaF_2_, Si, and ZnS) were found to be similar, showing the same general spectral shape which is markedly different from the average reflection spectra (MirrIR and Al slides) (Fig. 3). It is clear from this figure that there is a difference in the ratio of the absorbance in the high wavenumber region (covering O–H, N–H, and C–H stretching regions between 3,600 and 2,800 cm^−1^) with regard to the low wavenumber region (covering fingerprint region between 1,700 and 1,100 cm^−1^): This ratio is evidently much higher in the reflection spectra compared with the transmission ones.

**Fig. 4:**
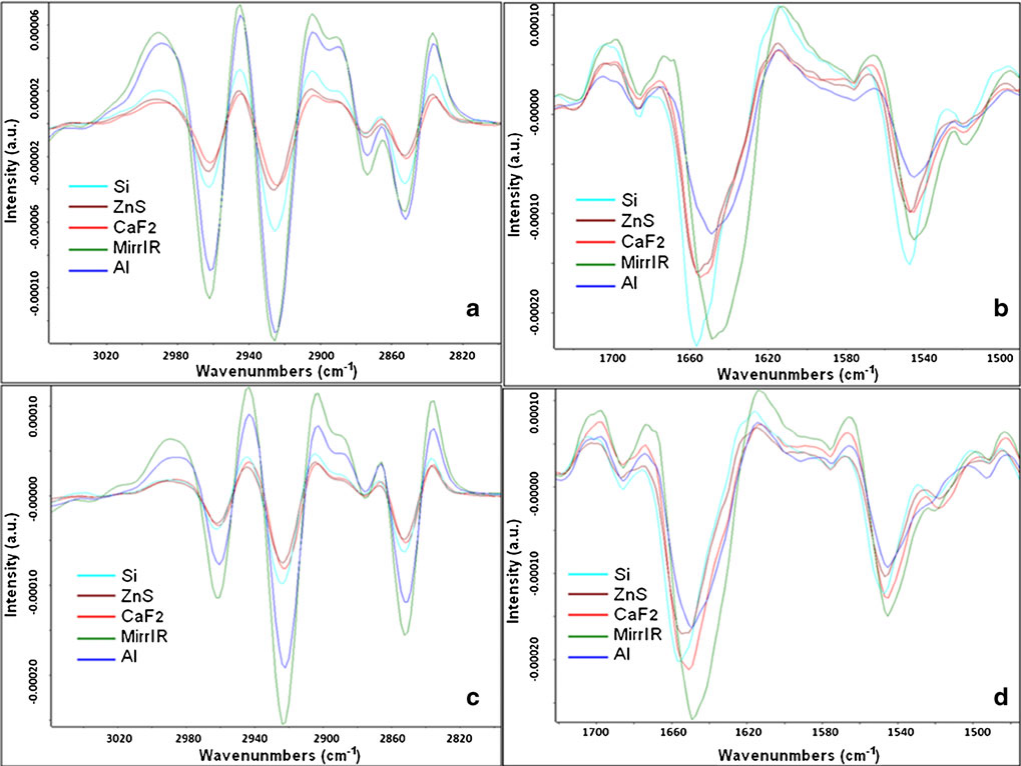
Second derivative average spectra of CHO-K1 (*upper graphs*) and DLD1 cells (*bottom graphs*) on different IR substrates. **a** Second derivative average spectra of CHO-K1 cells in the lipid region showing the higher intensity in R spectra (MirrIR and Al) with respect to T spectra (Si, ZnS, and CaF_2_); **b** second derivative average spectra of CHO-K1 cells in the protein region showing the shift between T and R spectra of about 7 cm^−1^ in the amide I peak; **c** second derivative average spectra of DLD1 cells showing again the same difference in the lipid region as for CHO-K1 cells; **d** second derivative average spectra of DLD1 cells in the protein region showing the shift between T and R spectra of about 5 cm^−1^ in the amide I peak. The color code for substrates is the same as in Fig. 5

Second derivative spectra plots of average spectra for CHO-K1 and DLD1 cells (Fig. 4) show a significant difference in intensity between transmission (T) and reflection (R) average spectra in the lipid region (3,050–2,800 cm^−1^) and a shift of, respectively, 7 and 5 cm^−1^ in the amide I region (1,700–1,600 cm^−1^). The amide I peak position for the average spectrum on each substrate in each cell line is illustrated in Table 1.

**Table 1:** Amide I peak position of the average cell spectra on each optical substrate

[cm^−1^]	Si	ZnS	CaF_2_	MirrIR	Al
CHO-K1	1,656.6	1,656.0	1,654.5	1,648.5	1,648.5
DLD1	1,655.5	1,654.6	1,652.0	1,648.7	1,648.7

The shift for the amide I peak between T set (Si, ZnS, and CaF_2_) and R set (MirrIR and Al) average spectra is about 7 and 5 cm^−1^ in CHO-K1 and DLD1 cells, respectively. There is also the slight shift toward the lower wavenumbers in the T spectra between Si, ZnS, and CaF_2_, respectively, which is correlated with their refractive index

#### Combined PCA of T and R cell spectra

**Fig. 5:**
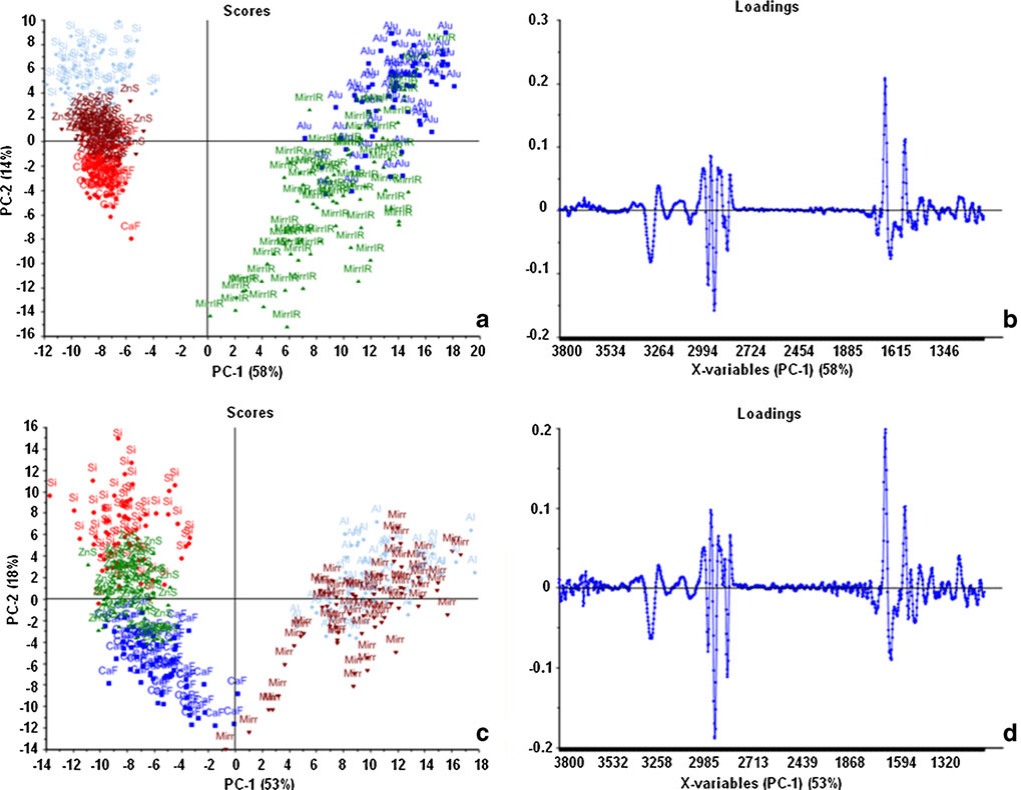
PCA and loadings of T and R sets for CHO-K1 (*upper graphs*) and DLD1 cells (*bottom graphs*). **a** PCA for raw spectra of CHO-K1 cells (*n* = 61 for Si, 67 for ZnS, 62 for CaF_2_, 70 for MirrIR, and 62 for Al). Spectra were second-derivative 17-points smoothing and SNV- normalized for the whole spectral range (3,800–1,100 cm^−1^) excluding CO_2_ (2,400–2,100 cm^−1^). T and R spectra are separated by PC1; **b** PC1 loadings showing the difference for the lipid and the protein: R spectra have more lipids and less protein than T spectra; **c** PCA for raw spectra of DLD1 cells (*n* = 61 for Si, 61 for ZnS, 61 for CaF_2_, 63 for MirrIR, and 66 for Al); **d** PC1 loadings showing the same difference for the lipid and the protein as in CHO-K1 cells

SNV was applied to second derivative spectra from CHO-K1 and DLD1 cells raw spectra. PCA was performed for the spectral region (3,800–1,100 cm^−1^) excluding the atmospheric CO_2_ region (2,400–2,100 cm^−1^). The results of the PCA analysis of T and R spectra for both cell types are shown in Fig. 5. The scores plots of PC1 versus PC2 for both cell types show a very similar distribution, with PC1 separating the reflection spectra from the transmission spectra and PC2 separating the transmission spectra by substrate. For this part of the discussion, only the variance explained by PC1 is discussed.

**Fig. 6:**
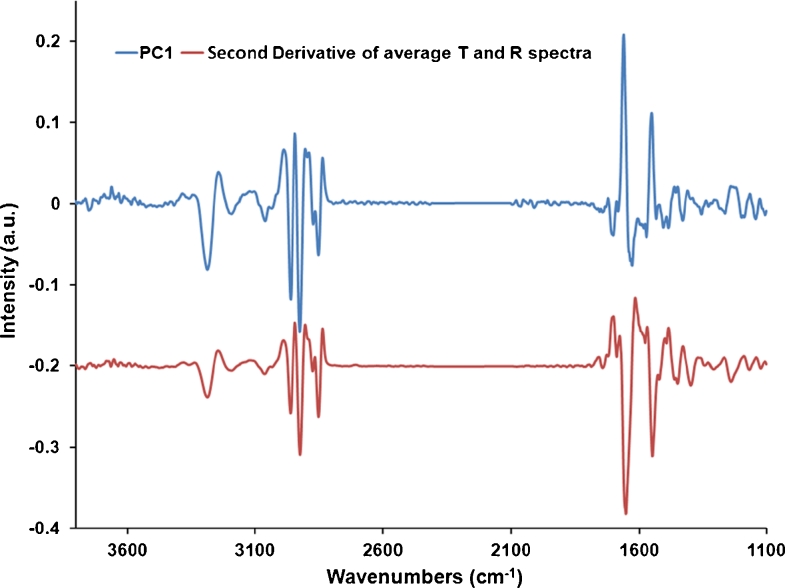
Combined PC1 loadings and second derivative of average spectra of T and R sets of CHO-K1 cells. The plots (shifted for clarity) are similar in the OH and lipid regions, but they are flipped (opposite way) over the protein region. Such difference between reflection and transmission spectra is due to the standing wave artifact

The PC1 loadings vectors for the two cell types (Fig. 5b for CHO-K1 and Fig. 5d for DLD−1) are almost identical, showing that the difference between the T and R spectra is consistent between the two cell types (major difference is in both lipid and protein regions). Comparing the CHO-K1 PC1 loadings vector to the mean spectrum of all the CHO-K1 cells (Fig. 6), the high wavenumber H-stretching region (3,600–2,800 cm^−1^) is the same in both spectra whereas the fingerprint region (1,700–1,100 cm^−1^) of the loadings vector is inverted compared with the mean. This pattern of the loadings vector, which effectively shows opposite signal sign respectively at high and low wavenumber, has been observed before in PCA analysis of cell R data, and it has been explained as an artifact due to an electric field standing wave [22, 23]. On reflection from a metal surface, incident and reflected light interfere creating a standing wave with a node at the surface. Given this boundary condition, thin samples (<1 μm) experience more of the electric field, and hence, show a relatively higher absorbance, for short wavelengths (λ/4 < 1 μm) than for longer ones. For these cell spectra, such artifact makes the ratio of the absorbance intensity of the high wavenumber H-stretching region (short wavelength) and the fingerprint region (long wavelength) vary dramatically depending on the thickness of the sample. This explains why the absorbance for the short wavelength H-stretching region was higher relative to the amide bands in the R data than the T data shown in Fig. 3. The different relationship between absorbance and thickness in reflection compared with transmission is the main reason for the shape of the loadings vector for PC1 for these two cell types, and it also explains why this is in common to both cell types.

Table 2 displays the average and standard deviation values of the PC1 scores for each substrate and for both cell types. These values reflect the intra-cluster variability of the scores values for each substrate and show that there is larger variation in PC1 for the R spectra than for the T spectra. In Fig. 5, T clusters especially for CaF_2_ and ZnS were more compact in both cell lines than the R sets, i.e., the spectra are closer to each other. The variation in the MirrIR spectral sets is higher than in other substrates for both cell lines. The spreading of CHO-K1 reflection spectra along PC1 for the MirrIR substrate is due to a greater range of thicknesses on MirrIR than on aluminum as determined from the overall absorbance values for the cells. This would make the spectra of the cell population on MirrIR be affected more by the standing wave artifact. The absence of this trend in the DLD1 cells potentially indicates a difference in the way the CHO-K1 cells grow on MirrIR compared with the DLD1.

**Table 2:** PC1 scores for CHO-K1 and DLD1 cells on all substrates

	CHO-K1	DLD1
PC1 scores	PC1, average ± SD	PC1, average ± SD
Si	−8.3 ± 1.8	−8.0 ± 2.1
ZnS	−7.8 ± 1.1	−7.6 ± 1.5
CaF_2_	−7.5 ± 0.8	−5.6 ± 2.2
Al	13.8 ± 2.5	10.4 ± 3.0
MirrIR	9.2 ± 3.7	9.5 ± 3.7

The standard deviation of PC1 scores on each substrate shows that the distribution variability of the clusters of cells on the reflective substrates is higher than the one on transmissive substrates (MirrIR highest variability for both types of cells)

#### PCA of the T cell spectra

**Fig. 7:**
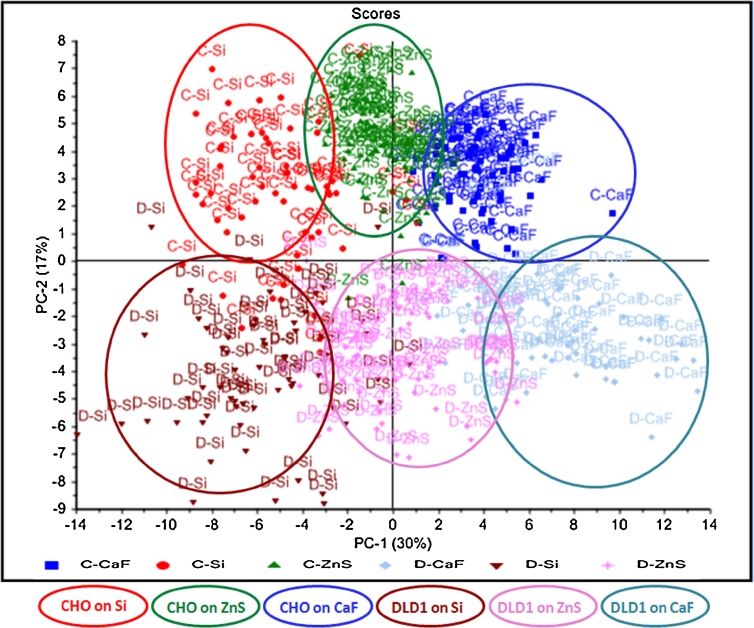
PCA of T spectra for CHO-K1 and DLD1 cells. PCA for raw transmission spectra of CHO-K1 cells (*n* = 61 for Si, 67 for ZnS, and 62 for CaF_2_) and DLD1 cells (*n* = 61 for Si, 61 for ZnS, and 61 for CaF_2_). Spectra were second-derivative 17-points smoothing and SNV- normalized for the whole spectral range (3,800–1,100 cm^−1^) excluding CO_2_ (2,400–2,100 cm^−1^). The separation among substrates is given by PC1 (30 %), in the order from *left to right* Si, ZnS, and CaF_2_, common to both cell lines. The separation of cell type is done by PC2 (17 %) where the *upper* set is for the CHO-K1 cells and the *lower* set is for DLD1 cells

**Fig. 8:**
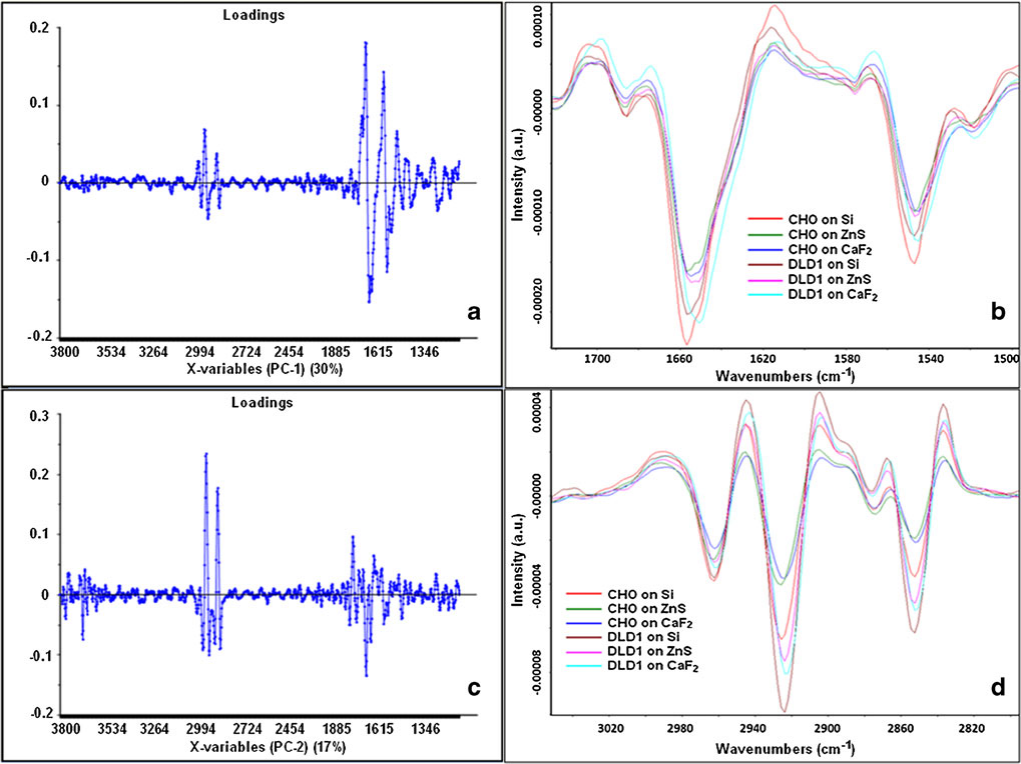
Explicative graphs for PCA in Fig. 7. **a** Loadings of PC1 making the separation of the substrate type suggesting there is a shift in amide I region; **b** second derivative graph of average spectra on the transmission IR optical substrate (Si, ZnS, and CaF_2_) in the protein region showing the slight shift of amide I peak (for peak position on each substrate refer to Table 1); **c** loadings of PC2 making the separation of cell type suggesting a difference in lipid intensity between CHO-K1 and DLD1; **d** second derivative graph of average spectra on the transmission IR optical substrate in the lipid region showing that DLD1 cells have higher lipid content than CHO-K1 cells

Having explained the difference between the reflection and transmission spectra, the PCA analysis was repeated focusing only on the transmission data for both cell lines grouped together (Fig. 7). PCA was performed for the spectral region (3,800–1,100 cm^−1^) excluding the CO_2_ region. Separation of window types (Si, ZnS, and CaF_2_) was done by PC1 (30 %) for both cell lines, and separation of cell lines between CHO-K1 and DLD1 was done by PC2 (17 %). The loadings of PC1 and PC2 are shown in Fig. 8. The loadings vector of PC1 (Fig. 8a) shows a difference in the lipid and the protein regions. Figure 8b shows the second derivatives of average spectra on T substrates in the amide region, and the amide I peak positions are given in Table 1. The amide I peak is different in each cell line for each window in the T sets. The peak shifts could be due to a genuine biochemical or morphological difference between cells grown on different materials, but the variation of the peak position is also consistent with the trend in refractive index value of the substrate (decreasing order of refractive index at wavelength around 5 μm—Si, 3.42; ZnS, 2.25; and CaF_2_, 1.4) suggesting that the shifts may be, in fact, due to an optical artifact. For both cell lines, the separation of the samples by PC1 shows the same order of clusters by substrate type. The analysis of an optical effect which can explain the artificial peak shift is detailed in the next section.

The loadings vector of PC2 (Fig. 8c) shows a difference in lipid absorption between the two cell lines as illustrated in the graph of the second derivatives for the average spectra (Fig. 8d): Here the absorbance of DLD1 cell spectra is higher than in CHO-K1 cell spectra, thus suggesting higher lipid content in DLD1 cells. This distinction between cell lines is consistent across all substrate materials and therefore clearly reflects a true biochemical difference. It is unlikely that using different growth media in the cell culture process could influence the separation of the two cell lines. Harvey et al. [24] studying the IR spectral signatures of different prostate cell lines confirmed that different growth media used for culturing the cells did not significantly influence the chemometric discrimination. This was not investigated in the present work as differentiation between the two cell lines CHO-K1 and DLD1 is not the main purpose.

### Reflection loss and optical artifact in cell transmission spectra

It is clear from Fig. 7 that PC1 scores discriminate the cell spectra mostly by their different substrates. CHO-K1 and DLD1 are two separate groups, but they are distributed along the PC1 axis depending on the Si, ZnS, and CaF_2_ material substrate, in order of decreasing refractive index.

The IR experiments in transmission had the sample illuminated through the substrate. It is expected that reflection losses related to the relative refractive indices at the substrate–sample interface play a major role. However, there are other interfaces—namely air–substrate and sample–air—whose optical contributions have to be considered.

Following a “detailed balance” of the measured IR beam intensity (I), the first interface encountered is air-substrate: This gives a constant reflection loss between background and sample measurements and so has no effect on the absorbance spectrum. The second interface (substrate–sample) is characterized by a reflectance R_12_, and the incoming IR beam is then attenuated by a factor (1-*R*_12_). Finally, the sample–air interface brings another reflection loss (1-*R*_23_). In total, ignoring multiple reflections, the transmission experimentally measured T* is related to the true sample transmission *T* by:

1 $$ {T^{*}}=T\left( {1-{R_{12 }}} \right)\left( {1-{R_{23 }}} \right) $$

Equation 1 ignores an additional reflection loss at the substrate–air interface when measuring the “background” intensity (*I*_0_) since this has no wavelength-dependent effect. The measured absorbance A* and the true absorbance *A* are related by:

2 $$ {A^{*}}=A-log\left( {1-{R_{12 }}} \right)-log\left( {1-{R_{23 }}} \right) $$

The key optical parameter is the refractive index (real part) *n*, more precisely, the ratio between the refractive index of the materials at the substrate–sample interface, *n*_12_ = *n*_1_/*n*_2._ Fresnel’s equations give the total reflectivity *R*_12_ as function of *n* (*s* polarization parallel, *p* normal to the surface):

3 $$ {R_s}={{\left( {\frac{{{n_{12 }}\cos {\theta_i}-\sqrt{{1-{{{\left( {{n_{12 }}\sin {\theta_i}} \right)}}^2}}}}}{{{n_{12 }}\cos {\theta_i}+\sqrt{{1-{{{\left( {{n_{12 }}\sin {\theta_i}} \right)}}^2}}}}}} \right)}^2}{R_p}={{\left( {\frac{{{n_{12 }}\sqrt{{1-{{{\left( {{n_{12 }}\sin {\cos {\theta_i}}} \right)}}^2}}}-{\cos_1}}}{{{n_{12 }}\sqrt{{1-{{{\left( {{n_{12 }}\sin {\theta_i}} \right)}}^2}}}+{\cos_1}}}} \right)}^2} $$

where *θ*_i_ is the incident angle of the beam (to the normal). The total reflectivity is then:

4 $$ {R_{\mathrm{tot}}}={{{\left( {{R_{\mathrm{s}}}+{R_{\mathrm{p}}}} \right)}} \left/ {2} \right.} $$

**Fig. 9:**
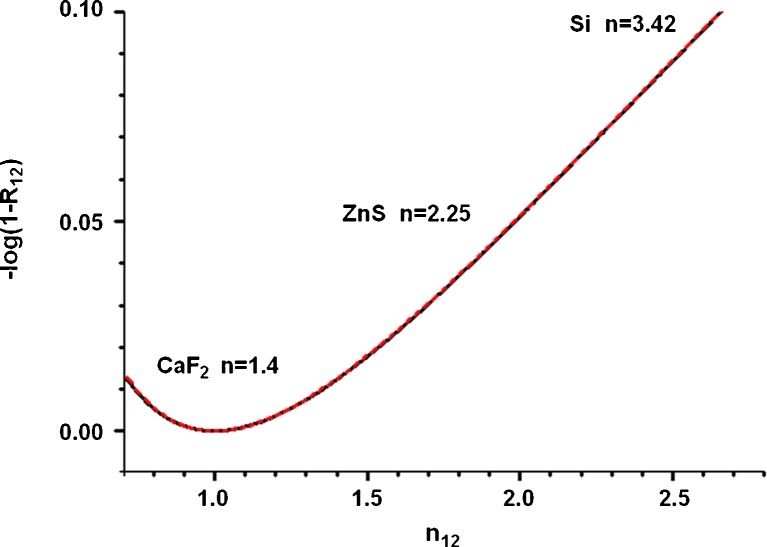
Reflection loss as function of the refractive index ratio between substrate (*1*) and sample (*2*). The *red line* refers to the 12° and the *black* to the 30° incident beams as per ×36 objective setup. An average value of the organic material refractive index has been used, namely *n*_2_ = 1.4

The IR microscope set up used a 36× cassegrain condenser, giving a range of angles for the IR beam onto the bottom surface between 12° and 30°. A generalized plot of the nonlinear relation between −log(1-*R*_12_) and *n*_12_ is shown in Fig. 9: Over this range, the Fresnel reflectivity is essentially independent of angle. However, the major conclusion from Fig. 9 is that reflection losses due to a change of *n*_12_, i.e., at the substrate–sample interface, accounts via Eq. 2 for a systematic variation between the true sample absorbance A and the measured one A* of up to about 0.1 (for Si, major refractive index difference). When sample and substrate have closer value of *n*, this systematic variation decreases (e.g., ZnS) or becomes zero (CaF_2_). With reference to Fig. 7 where the same cell line measured in transmission is classified as function of the substrate material, there is a clear correlation between the PC1 score and this reflection loss. Specifically, the order cells group along PC1 in Fig. 7—respectively Si, ZnS, and CaF_2_—corresponds to the amount of the reflection loss shown in Fig. 9. In brief, the “detailed balance” quantifies the reflection loss at the substrate–sample interface and its scaling with the optical substrate refractive index. It is now necessary to evaluate the spectral dependence of the reflection losses.

The loadings vector of PC1 gives the spectral difference between substrates. The same can be done analytically from Eq. 2 by taking the differential absorption spectra between substrates: for example, with respect to CaF_2_ which has the closest refractive index to the sample (*n*_12_ ~ 1):

5 $$ {A^{*}}_{\mathrm{Substrate}}-A_{\mathrm{CaF}2}^{*}=-\log {{\left( {1-{R_{12 }}} \right)}_{\mathrm{Substrate}}}-\log {{\left( {1-{R_{12 }}} \right)}_{\mathrm{CaF}2}} $$

Cells on CaF_2_ have negligible reflection loss (Fig. 9), thus the valid approximation:

6 $$ {A^{*}}_{\mathrm{Substrate}}-A_{\mathrm{CaF}2}^{*}\approx -\log {{\left( {1-{R_{12 }}} \right)}_{\mathrm{Substrate}}} $$

**Fig. 10:**
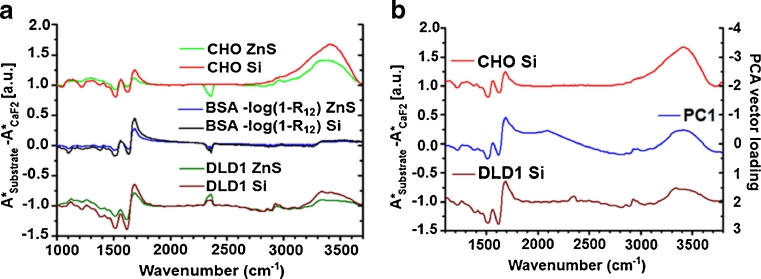
Reflection loss and optical artifact in transmission spectra. **a** Spectral difference of CHO-K1 and DLD1 cell absorption spectra on respectively ZnS and Si to the corresponding ones on CaF_2_. Cells lines are offset for clarity. The reflection loss is calculated from the specular reflection from a BSA thick sample. **b** *Left* scale: IR absorption difference of CHO-K1 and DLD1 cell average spectra on Si substrate. *Right* scale: PC1 loading vector after double integration and baseline subtraction

Here, the reflection loss could be calculated via Eq. 3 knowing the refractive index spectrum of the cell samples. This cannot be easily measured, so the refractive index of a homogenous and similar biological material, bovine serum albumin (BSA), was derived from an IR reflectivity measurement at the sample–air interface. Having a similar absorbance spectrum to a cell, BSA closely reproduces all the major spectral features of biological samples with a similar refractive index spectrum. The results are shown in Fig. 10, which is the graphical representation of Eq. 6.

Figure 10a shows the measured A*_Substrate_ − A*_CaF2_ difference spectra for the cell samples along with the calculated −log(1-*R*_12_) spectra for BSA on each substrate, using the BSA–substrate relative refractive indices and Fresnel’s equations. Along the entire mid-IR spectral range, there is a close match between the cell absorbance difference A*_Substrate_ − A*_CaF2_, for both CHO-K1 and DLD1 cell lines and the reflection loss estimation −log(1-*R*_12_). Also, the reflection loss amplitude scales correctly with the substrates shown, i.e., lower amplitude for ZnS and higher for Si. This confirms that the reflection loss and related optical artifact is clearly responsible for the substrate-dependent spectral changes.

Finally, the measured IR spectra difference needs to be compared with the findings from the principal component analysis on transmission data, namely PC1 loading vector. The substrate type discrimination based on PC1 was performed on the second derivative spectra, thus the actual PC1 loading vector has been integrated twice^1^ to recollect the absorbance information. The result is plotted in Fig. 10b, together with the experimental absorbance difference of cells on Si versus CaF_2_ substrate. Again, there is a striking match between the loading vector of PC1 and the difference spectra A*_Substrate_ − A*_CaF2_ across the whole spectral range.

The derivate-like signal of this reflection loss is responsible for the shifting of the amide I peak in the T data shown in Table 1, but it also explains the shift between the T and R spectra and smearing of the reflection data across PC2 in the combined PCA of R and T data of Fig. 5a, c. In an IR reflection measurement, the signal consists mainly of the light transmitted through the sample and reflected off the mirror substrate (transflected), but there is also a detected reflection at the sample top surface [25] which is the −log(1-*R*_23_) term in Eq. 2. The top-surface reflected light has the same spectral features as the transmission artifact because both depend on the refractive index of the sample. It is the detection of this light that causes the peak shift between the T and R data. Further to this, cells of different thickness would show a different ratio of reflection and transflected signals, since the latter has a linear dependence with optical path in the sample via the absorbance while the former does not change. In practice, it is this that can cause the wide spreading of the reflection data in the combined PCA analysis as shown in Fig. 5.

Several optical artifacts have been treated before. Bassan et al. [25] discussed dispersion artifacts in transflectance IR data due to sample optical density and index variation. Attention has been particularly given in the IR literature to the Mie scattering artifact and software correction for single-cell analysis both in non-resonant [26] and resonant formalism [27], with dispersive effects shown through the scattering amplitude dependence on the relative refractive index change between scattering object and surrounding, e.g., nucleus and cell cytoplasm. Miljkovic et al. [28] have revised and applied the phase correction method onto general line shape distortions in IR spectra. All these approaches rely on iterative algorithms where phenomenological parameters have to be optimized, e.g., the IR signal is fitted in terms of transmission and reflection components [25], or refractive index and sphere radii in the extended multiplicative signal correction [26], or the convergence from the reference IR spectrum [27], or the best-phase angle [28]. In this work, with no free parameters, our model can quantitatively account for the reflection losses in IR transmission data using Fresnel’s equations and via the Kramers–Kronig transformation of experimental reflectivity data.

### Correction of the transmission spectra

For accurate spectra and absolute IR peak positions for cells on different transmission substrates, it would be necessary to correct the IR absorbance for the reflection losses as accounted by Fresnel’s equations.

The analytical correction can be outlined as follows:

First, the refractive index spectrum of the sample is obtained, which for practical purposes could be via Kramers–Kronig transformation of the reflectance IR spectrum of a thick sample of the same cell line(s) used in the experiment. Ideally, an IR measurement on CaF_2_ substrate will avoid any back-reflected components from the sample–substrate interface.

Secondly, the reflection losses from the sample–substrate and sample–air interfaces, i.e., −log(1-*R*_12_) and −log(1-*R*_23_) are calculated, using Fresnel’s equations (Eqs. 3 and 4), including the sample–substrate and sample–air relative refractive index spectra.

Such reflection losses are finally subtracted from the raw absorbance data before normalization, since the losses are independent of sample thickness.

Any further treatment of the data to account for, e.g., Mie scattering can then be performed as required.

## Conclusion

The first objective of this work was to assess the cell viability on some of the most used IR substrates. The results show that BaF_2_ and ZnSe are not suitable for cell growth due to low cell viability. Three other IR transparent materials—Si, ZnS, and CaF_2_—are biochemically compatible for cell growth as they proved a high percentage (above 90 %) of viability and similar morphology of cells to standard polystyrene culture flasks. MirrIR- and Al-coated glass slides typically used for IR reflection mode are suitable for in situ cell culture.

In the analysis of IR spectra of single cells in transmission and reflection on the IR materials above and for two cell lines, no substrate-induced biochemical variations could be revealed. IR data for single cells in transflectance confirm that the standing wave artifact plays the major role, and such absorption spectra are affected by a dramatic non-linear dependence in absorption with sample thickness in between the fingerprint and the H stretching region, respectively below and above 2,000 cm^−1^.

In transmission, the IR spectral discrimination is dominated by an optical artifact due to the substrate reflectivity, which depends on the relative refractive index ratio sample–substrate. This is also the cause of the minimal shift of main absorption bands (such as the amide I) to higher wavenumbers with increasing substrate refractive index. A model based on Fresnel’s equations explaining in detail the phenomenon and quantifying the effect in the mid-IR spectral region is proposed and compared well with the experimental data. Out of the three transmission substrates that could be used for growing cells for FTIR microspectroscopy, CaF_2_ has the less reflective loss at the substrate–sample interface. If the interest is in the lower wavenumbers, e.g., DNA-RNA region below 1,000 cm^−1^, ZnS is preferable and offers a wider transmission range at the cost of some more spectral distortion of this kind. In general, for comparative cells studies by IR microspectroscopy in transmission when IR peak position is not crucial, it is sufficient that all samples are measured on the same substrate type. For accurate and absolute peak positions, it would be necessary to correct the transmission spectra for the reflection losses, for example, via Fresnel’s equation as used in this work. These results may help the IR biomedical community to make a proper choice of the substrate for cells experiments, ideally in view of a standardization of the FTIR protocol for all researchers interested in studying cells by FTIR microspectroscopy.

## Abbreviations

Al: Aluminum
BaF_2_: Barium fluoride
BSA: Bovine serum albumin
CaF_2_: Calcium fluoride
DAPI: 4′,6-Diamidino-2-phenylindole
FBS: Fetal bovine serum
FTIR: Fourier-transform infrared
NaCl: Sodium chloride
MCT: Mercury cadmium telluride
MIRIAM: Multimode InfraRed Imaging And Microspectroscopy
PI: Propidium iodide
Si: Silicon
Si_3_N_4_: Silicon nitride
SR: Synchrotron radiation
XRF: X-ray fluorescence
ZnSe: Zinc selenide
ZnS: Zinc sulfide

## Footnotes

1 This procedure is preferred since integration does not enhance numerically the spectral noise.
